# A prospective, open-label, randomized clinical trial to evaluate the efficacy and safety of remimazolam in patients undergoing EBUS-TBNA: REST trial design

**DOI:** 10.1186/s12890-024-03067-w

**Published:** 2024-05-17

**Authors:** Hee Yun Seol, Kyung Soo Hong, Jong Geol Jang, Seong Mi Moon, Sun-Hyung Kim, Jun Yeun Cho, Bumhee Yang, Seonok Kim, Chang-Min Choi, Wonjun Ji, June Hong Ahn

**Affiliations:** 1 Department of Internal Medicine, Pusan National University School of Medicine, Busan, Republic of Korea; 2 Division of Pulmonary and Critical Care Medicine, Research Institute for Convergence of Biomedical Science and Technology, Pusan National University Yangsan Hospital, Yangsan, Republic of Korea; 3 Division of Pulmonology and Allergy, Department of Internal Medicine, College of Medicine, Yeungnam University and Respiratory Center, Yeungnam University Medical Center, 170 Hyeonchung-Ro, Namgu, Daegu, 42415 Republic of Korea; 4 Division of Pulmonary and Critical Care Medicine, Department of Internal Medicine, Chungbuk National University Hospital, Chungbuk National University College of Medicine, Cheongju, Republic of Korea; 5 Department of Clinical Epidemiology and Biostatistics, Asan Medical Center, University of Ulsan College of Medicine, Seoul, Republic of Korea; 6 Department of Pulmonary and Critical Care Medicine, Asan Medical Center, University of Ulsan College of Medicine, 88 Olympic-Ro 43-Gil, Songpa-Gu, Seoul, 05505 Republic of Korea

**Keywords:** Sedation, Remimazolam, Endobronchial ultrasonography, Protocol, Pulmonary medicine

## Abstract

**Background:**

Remimazolam is safe and effective for moderate sedation during flexible bronchoscopy, but its safety and efficacy during endobronchial ultrasound-guided transbronchial needle aspiration (EBUS-TBNA) remains undetermined. The REST trial (NCT06275594) will be a prospective randomized study of remimazolam in patients undergoing EBUS-TBNA with conscious sedation. The primary aim is to evaluate whether remimazolam is safe and effective for moderate sedation during EBUS-TBNA compared to real-world midazolam and on-label midazolam.

**Methods:**

The REST trial will recruit 330 patients from four university hospitals with mediastinal lesions suspected of being lung cancer who are eligible for EBUS-TBNA under moderate sedation. The participants will be randomized into groups using remimazolam, real-world midazolam, and on-label midazolam (US prescribing information dosage) to perform EBUS-TBNA for procedural sedation. The primary endpoint will be procedural success using composite measures.

**Discussion:**

The REST trial will prospectively evaluate the efficacy and safety of remimazolam during EBUS-TBNA under moderate sedation. It will provide information for optimizing sedation modalities and contribute to practical benefits in patients undergoing EBUS-TBNA.

**Trial registration:**

ClinicalTrials.gov (NCT06275594). Prospectively registered on 15 February 2024.

## Background

Real-time endobronchial ultrasound-guided transbronchial needle aspiration (EBUS-TBNA), developed for examining mediastinal lymph nodes, uses an ultrasonic probe at the end of a conventional flexible bronchoscope. Compared to a flexible bronchoscope, it has a greater diameter and requires repeat needle aspiration from mediastinal lymph nodes. Thus EBUS-TBNA is a relatively longer procedure than flexible bronchoscopy [1, 2].

Systematic EBUS-TBNA includes sampling suspected lymph nodes based on imaging studies and routine sampling of stations 4R, 4L and 7 (if the short axis ≥ 8 mm). The SCORE study showed that compared to positron emission tomography guided targeted EBUS, a systematic EBUS procedure followed by esophageal investigation using the same EBUS bronchoscope (EUS-B), increases the sensitivity for detecting mediastinal nodal metastasis in lung cancer patients by 9% [3]. Given that the EBUS scope has a greater diameter and more stations need to be sampled during systematic EBUS-TBNA staging, appropriate sedation is essential.

Midazolam (onset of action 3–5 min) is an important agent for moderate sedation; however, the active metabolite of midazolam has an elimination half-life of 1.8–6.4 h, which can prolong post-procedural sedation. Propofol is a potent intravenous sedative with a very short onset of action (15–40 s) and a very short half-life, allowing rapid recovery. However, it requires constant monitoring due to potential respiratory depression or hypotension without antidote medication [4, 5].

Remimazolam is a novel benzodiazepine used for intravenous sedation. It has an ultra-short duration of action. It has been successfully used in procedural sedation and general anesthesia with fast onset and recovery times, high procedure success rates, and acceptable adverse profiles [5, 6]. Therefore, the benefits of remimazolam derived from its fast onset and recovery times warrant further examination in a prospective, controlled study. To date, no studies have evaluated the effectiveness and safety of remimazolam during EBUS-TBNA. The REST trial will evaluate whether remimazolam provides benefits in performing EBUS-TBNA compared to real-world midazolam, or on-label midazolam.

## Methods/design

### Study design and inclusion criteria

**Fig. 1:**
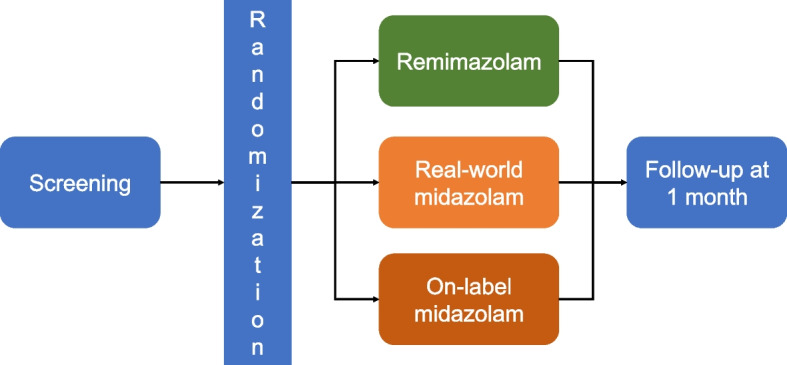
Schematic outline of the study design

The REST trial will be a multicenter, open label, randomized clinical trial that will enroll patients undergoing EBUS-TBNA under conscious sedation to evaluate the efficacy and safety of remimazolam compared to real-world midazolam or on-label midazolam (Fig. 1). After providing informed consent, participants will be recruited from four institutions across South Korea: Yeungnam University Hospital, Asan Medical Center, Pusan National University Yangsan Hospital, and Chungbuk National University Hospital. The study will recruit participants from 1 April 2024 until 1 February 2025. Participants will be followed until 1 month after the procedure.

All consecutive patients over 18 years of age who are candidates for EBUS-TBNA due to suspected malignancy, with saturation pulse oximetry (SpO_2_) ≥ 90% in ambient air or with no more than 2 L/min oxygen support are eligible for enrollment. The exclusion criteria are patients undergoing simultaneous radial EBUS or electromagnetic navigation bronchoscopy; American Society of Anesthesiologists (ASA) score 4 or higher; Mallampati score 4; body mass index (BMI) < 18.4 or > 30.0; known allergic reaction to benzodiazepines, flumazenil, opioids, naloxone, or a medical condition affected by these agents; EBUS-TBNA performed outside the bronchoscopy unit; chronic kidney disease requiring dialysis; and patients with a current active acute infection or uncontrolled chronic inflammatory lung disease.

### Randomization, data collection, and management

After providing informed consent, participants will be randomized centrally 1:1:1 to the remimazolam, real-world midazolam, or on-label midazolam arms after stratification by study site, age ≥ 60 vs. < 60 years, and BMI (> 23 vs. ≤ 23 kg/m^2^).

Randomization will be performed using a web-based electronic case report form (eCRF) created by Procuratio®. The assignment process will not involve the attending physicians. However, this study will be an open label study, so physicians will know what sedatives the patients are given. The enrolled participants will be evaluated for baseline characteristics, procedural profile, pathological results, and cough using a procedural convenience survey after study enrollment. The data to be collected are listed in Table 1. Complication profiles will be observed and recorded for up to 1 month after the procedure.

**Table 1:** Data to be collected from study participants

Baseline clinical characteristics
Age
Sex
Height
Body weight
ASA
Smoking status
Pack-years smoked
Past medical history
Pulmonary function tests
Concurrent anticoagulant and antiplatelet agent usage
Use of prophylactic antibiotics
Procedure characteristics
Sedative administration time and dosage
EBUS procedure start and end times
Type of EBUS scope used
Amount of oxygen administered
Requirement for flumazenil
Vital signs during the procedure
Cough and convenience surveys
Pathological diagnosis
Final diagnosis
Procedure complications

*ASA* American Society of Anesthesiologists, *EBUS* Endobronchial ultrasound

A web-based eCRF created by Procuratio® will be used to gather data. The eCRFs will be collected at the time of study enrollment and at a follow-up outpatient visit within 1 month after the procedure. The web-based eCRF will anonymize and secure all data. The enrollment procedure began in April 2024 with the goal of being completed by February 2025. The last round of data collection and follow-up is expected to be completed in March 2025.

### Sedative, and procedural protocols

Before bronchoscopy, the oropharynx will be sprayed 10 times (10 mg/puff) with 10% lidocaine spray solution. Using a bronchoscope, 1% lidocaine solution will be administered to the vocal cords, mid-trachea, and both major bronchi. Additional lidocaine will be administered if requested by the bronchoscopist. The patient will be given oxygen at 5 L/min via nasal cannula.

An initial dose of 25–50 µg fentanyl and a single intravenous dose of 5.0 mg remimazolam (ASA I-II) or 2.5 mg remimazolam (ASA III) will be administered in the remimazolam arm. For the real-world midazolam arm, 2–3 mg midazolam will be administered with a maximum dose 7.5 mg. For on-label midazolam, 1.75 mg (< 60 years old and healthy) or 1.0 mg (≥ 60 years old or debilitated/chronically ill) midazolam will be administered to a maximum dose of 7.5 mg. The detailed sedation protocols are presented in Table 2.

**Table 2:** Sedative protocols in the three arms

Arm	Intervention
Remimazolam	ASA I–II: initial dose 5 mg, top-up dose 2.5 mg, interval ≥ 2 min ASA III: initial dose 2.5 mg, top-up dose 1.25 mg, interval ≥ 2 min Maximum remimazolam dose, no limit Fentanyl: 25–50 μg, interval ≥ 5 min, maximum dose 200 μg
Real-world midazolam	Initial dose 2–3 mg, top-up dose 0.5–1 mg, interval ≥ 2 min Maximum midazolam dose 7.5 mg Fentanyl: 25–50 μg, interval ≥ 5 min, maximum dose 200 μg
On-label midazolam	< 60 years old and healthy: initial dose 1.75 mg, top-up dose 1.0 mg, interval ≥ 2 min ≥ 60 years old or debilitated/chronically ill: initial dose 1.0 mg, top-up dose 0.5 mg, interval ≥ 2 min Maximum midazolam dose 7.5 mg Fentanyl: 25–50 μg, interval ≥ 5 min, maximum dose 200 μg

*ASA* American Society of Anesthesiologists

Bronchoscopy will be started when an adequate sedation Modified Observer’s Assessment of Alertness/Sedation Scale (MOAA/S ≤ 3) score is achieved. If sedation is insufficient after the first dose of trial medication, patients can receive a maximum of five additional remimazolam doses in any 15 min window or a maximum of three additional doses of midazolam in any 12 min window in the real-world and on label midazolam arms at intervals of ≥ 2 min. Top-up doses of fentanyl (25–50 μg) at intervals of ≥ 5 min are permitted in all three arms, until adequate analgesia is achieved or a maximum of 200 μg has been administered. If there is still insufficient sedation to start the procedure, treatment failure is declared and rescue sedation is administered using drugs from the treatment group under the supervision of a respiratory physician without anesthesia personnel. During the procedure, all patients will be monitored via electrocardiogram, noninvasive blood pressure, oxygen saturation, and respiratory rate every 5 min. EBUS-TBNA procedures will be performed using the Olympus (BF-UC260FW) or EB‐1970UK (Pentax) by pulmonologists with at least 5 years of experience with EBUS-TBNA. The operator’s biopsy method will be respected without clear regulations and EBUS profiles will be recorded, including lymph node biopsy results by station, final diagnosis, and complication.

### Sample size calculation

In the REST trial, expected procedural success of remimazolam group was 80%, those of real-world midazolam (control 1) was 60%, and those of on-label midazolam (control 2) was 40% [4, 7]. Therefore, two sided multiple comparison analysis was planned to test whether the procedural success rate of remimazolam group was significantly different from the two midazolam group. We accepted the 0.025 significance level under Bonferroni correction for multiple comparison. The sample size of each groups was 96 and the corresponding powers of each of the tests were 0.8. Based on an assumed 10% drop-out rate of patients after randomization, we aim to include a total of 330 patients.

### Assessment of study outcomes

The primary objective is to compare the procedural success with remimazolam, real-world midazolam, or on-label midazolam using a composite measure. Secondary endpoints include the times until the procedure is started and the patient is fully alert after the procedure and the total procedure and recovery times. The requirement for flumazenil and total fentanyl dose are included. A cough visual analogue scale (VAS) and procedure convenience VAS measured by the patient, physicians performing the procedures, and nurses in the bronchoscopy room are also included. For safety, the vital signs will be recorded. All participants will be followed-up 1 month after the procedure to evaluate the presence of complications. The detailed definitions of the outcomes are presented in Table 3.

**Table 3:** Definitions of study outcomes

Primary endpoint: Success of the EBUS procedure
Success of the EBUS procedure via a composite measure
-Completion of the EBUS procedure
-No requirement for a rescue sedative
-For remimazolam, no more than five top-up doses of the study medication required within any 15 min period; for midazolam, no more than three top-up doses required in any 12 min window
Secondary endpoints
Time to starting the procedure after administering the first dose of the study medication: the time from the administration of the first dose to the start of the procedure (MOAA/S ≤ 3)
Time taken to achieve full alertness after the procedure: the time from the end of the procedure to the first of three consecutive MOAA/S scores = 5
Requirement for flumazenil during the procedure: if an MOAA/S score 5 is not reached after bronchoscopy, flumazenil will be administered. The total amount of flumazenil administered during the procedure is recorded
Requirement for fentanyl during the procedure: the total amount of fentanyl during the procedure
Scales of cough and procedural convenience: for the cough and procedural convenience visual analogue scales (VAS), patients mark a point on a straight line corresponding to their perception of the severity of the cough, discomfort, and inconvenience. VAS ranges from 0 to 100, with 0 representing the minimal severity and 100 representing the maximal severity
Changes in vital signs and oxygen saturation during the procedure: the changes in vital signs (blood pressure, pulse rate, and respiration rate) and oxygen saturation assessed every 5 min
Complications related to the procedure
Pneumothorax
Grade 1: asymptomatic; clinical or diagnostic observations only; intervention not indicated Grade 2: symptomatic; intervention indicated (e.g., thoracic tube insertion without pleurodesis) Grade 3: pleurodesis or operative intervention indicated; hospitalization indicated Grade 4: life-threatening consequences; urgent intervention indicated Grade 5: death
Bleeding
Mild: If controlled by the application of cold saline or epinephrine solution Moderate: Needs a systemic agent (intravenous tranexamic acid), argon plasma coagulation, or laser ablation Severe: Needs a transfusion, bronchial artery embolization, unplanned endobronchial blockade (balloon), intubation or other life-threatening consequences

*EBUS* Endobronchial ultrasound, *MOAA/S* Modified Observer’s Assessment of Alertness/Sedation Scale scores

## Ethics approval

The REST trial will be conducted in accordance with the principles of Good Clinical Practice and the tenets of the Declaration of Helsinki. The Institutional Review Board of each participating institution have already approved the study protocol: Yeungnam University Hospital (YUMC 2023–12–017), Asan Medical Center (S2023–2655), Pusan National University Yangsan Hospital (11–2023–024), and Chungbuk National University Hospital (2023–11–003–003). Informed consent will be obtained from all participants. The study is registered at ClinicalTrials.gov (NCT06275594).

### Trial status

REST Trial is ongoing now. Enrollment began in April 2024 and is currently in progress with the aim of completing enrollment by March 2025. Final collection of data and formal analysis is planned at mid-2025.

## Discussion

Proper sedation plays a crucial role in EBUS-TBNA procedures. It ensures patient comfort, reduces anxiety, and minimizes movement during the procedure, which is important for both the accuracy and safety of the biopsy. Furthermore, it can affect the overall patient experience. Patients who receive adequate sedation often report less discomfort, have less recall of the procedure, and show a greater willingness to undergo the procedure again if needed [8]. Various sedatives can be used for EBUS-TBNA to ensure patient comfort and procedure efficacy. Sedatives and opioids are frequently combined to achieve optimal sedation and analgesia. Commonly used sedatives include midazolam, ketamine, and propofol. Midazolam, commonly used for its anxiolytic and amnesic effects, is often administered in combination with fentanyl for EBUS-TBNA procedures. However, it has some disadvantages; while effective as a sedative, its onset may be delayed and its duration may be extended beyond the desired therapeutic window [9]. This is particularly evident in the elderly or patients with hepatic dysfunction, in whom the pharmacokinetics of midazolam are altered, leading to reduced metabolic clearance and consequently prolonged drug action. Such pharmacodynamic variability necessitates cautious administration and vigilant monitoring to mitigate the risks associated with protracted sedative effects [10].

Remimazolam is a recently developed ultra-short-acting benzodiazepine sedative [11]. Recent meta-analyses and studies have reported its usefulness for procedural sedation [12]. Its safety and effectiveness at providing sedation during bronchoscopy has been studied [4, 13]. Its unusual metabolism results in a quick onset and brief duration of effect [14]. In clinical trials, it has been found to offer efficient sedation with a superior profile to midazolam during bronchoscopy [4]. It achieves sleepiness rapidly without causing major changes in hemodynamic stability. It has also been linked to the reduced occurrence of bradycardia or hypotension compared to dexmedetomidine [15]. Compared to conventional bronchoscopy, EBUS-TBNA takes longer and involves direct contact with and irritation of the airway. Therefore, ensuring proper sedation is crucial. However, despite the advantage of the action time of remimazolam, no research has examined it in EBUS-TBNA.

Maintaining appropriate sedation during procedures is difficult, particularly when trying to maintain it at a moderate level. Without proper sedation, performing procedures becomes challenging, and more midazolam than the recommended dose may be used. In a recent study that compared the efficacy of remimazolam with midazolam dosed according to real-world medical practice and on-label prescribing information, remimazolam had similar effects to real-world midazolam for some parameters and was comparable to on-label midazolam in others [16]. In this trial, we will compare remimazolam with two different midazolam dosages to enhance its practical applicability in actual clinical environments.

The study participants will be drawn from four tertiary hospitals in South Korea, with minimal limitations on procedure settings. The study will standardize the method of local anesthesia and oxygen supply, while maintaining flexibility in other procedural areas to accommodate the specific situations at each hospital. Given that the study will involve three distinct cohorts within each facility, the results should be particularly applicable to real-world data.

The major limitation of this study is its open-label design, which lacks blinding and can introduce bias and affect the interpretation of results. To minimize observer bias and ensure objectivity in an open-label study, the cough visual analogue scale (VAS) and procedure convenience VAS will be obtained from clinicians, nurses, and patients. In addition, other evaluation criteria will be standardized and clearly defined.

In conclusion, the REST trial will be a unique, investigator-initiated trial that aims to identify the efficacy and safety of remimazolam in performing EBUS-TBNA, particularly in procedural sedation in adults outside the operating room. This study should determine the efficacy of remimazolam compared to midazolam and improve our understanding of this sedative for use in EBUS-TBNA. The trial will provide important information on the optimal usage of sedatives for EBUS-TBNA. The study outcomes will also be instructive for designing future comparative studies of EBUS-TBNA.

## Data Availability

The datasets generated and analyzed during the current study will be available from the corresponding author on reasonable request.

## Abbreviations

ASA: American Society of Anesthesiologists
BMI: Body mass index
EBUS-TBNA: Endobronchial ultrasound-guided transbronchial needle aspiration
eCRF: Electronic case report form
MOAA/S: Modified Observer’s Assessment of Alertness/Sedation Scale
VAS: Visual analogue scale
